# Calcium Sulfide Nanoclusters Trigger DNA Damage and Induce Cell Cycle Arrest in Non-Small-Cell Lung Adenocarcinoma Cells

**DOI:** 10.3390/ijms26041665

**Published:** 2025-02-15

**Authors:** María M. Figueroa Rosado, Kevin Muñoz Forti, Patricia Rodríguez-Rodríguez, Gerardo Arroyo-Martínez, Valerie J. Rodríguez-Irizarry, Abigail Ruiz-Rivera, Jailenne I. Quinones-Rodriguez, Pedro G. Santiago-Cardona, Olga M. Rodriguez Martinez, Sharilyn Almodovar, Miguel E. Castro, Edu Suárez Martínez

**Affiliations:** 1 Department of Biology, University of Puerto Rico, Ponce 00732, Puerto Rico; 2 Department of Clinical Anatomy, College of Osteopathic Medicine, Sam Houston State University, Conroe, TX 77304, USA; 3 Department of Basic Sciences, Ponce Health Sciences University, Ponce 00716, Puerto Rico; 4 BioEngineering Graduate Program, University of Puerto Rico, Mayagüez 00680, Puerto Rico; 5 Department of Immunology & Molecular Microbiology, Texas Tech University Health Sciences Center, Lubock, TX 79430, USA; 6 Department of Chemistry, University of Puerto Rico, Mayagüez 00680, Puerto Rico

**Keywords:** calcium sulfide, nanostructures, non-small-cell lung carcinoma

## Abstract

Lung cancer remains the most common malignancy independent of sex. Here, we focused on unraveling the molecular mechanisms of CaS nanoclusters inducing cytotoxicity by investigating DNA damage, the cell cycle, oxidative stress, and cellular repair mechanisms in non-small-cell lung carcinoma (NSCLC) cells compared to healthy lung fibroblasts. Our previous studies have demonstrated the therapeutic potential of calcium sulfide (CaS) nanostructures in skin and breast cancer models, leading to a significant reduction in cancer cell proliferation. However, how CaS nanoclusters enhance their therapeutic effects on cancer cells while minimizing damage to healthy cells remains unknown. Our results show that CaS nanoclusters, once dissociated into ${Ca}^{2+}$ and $H_{2}S$ in an acidic microenvironment, selectively allow extracellular calcium to enter, leading to an increase in free calcium entry, triggering oxidative stress and limiting DNA repair mechanisms in NSCLC. Furthermore, CaS nanoclusters selectively arrest NSCLC cells in the G0-G1 and S phases of the cell cycle without affecting healthy cells’ cycles. Here, we also show that the selective effects of CaS nanoclusters on lung adenocarcinoma are less likely to be regulated by intrinsic apoptotic or mitochondrial pathways. They are, rather, caused by an increase in Ca^2+^ and ROS, causing double-stranded DNA breakages. This selectivity for malignant cells is pH-dependent because it occurs in the acidic microenvironment characteristic of these cells. Overall, this is the first piece of evidence that CaS disrupts genomic stability, prevents the replication of damaged cells, and ultimately influences cell fate decisions such as cell cycle arrest or cell death including mitotic catastrophe and necroptotic simultaneous events.

## 1. Introduction

Lung cancer is the most common malignancy in both men and women. Non-small-cell lung cancer (NSCLC) accounts for 80–85% of diagnosed lung cancers. Diagnosed individuals have a 5-year survival rate of 50% after treatment and a recurrence rate of 35% to 50% when diagnosed at early stages of the disease [1,2,3]. First-line treatment options include radiotherapy, chemotherapy, and surgery. Patients with NSCLC are highly susceptible to developing secondary tumors that tend to be resistant to previously used treatments. The growing field of nanomedicine offers the promise of using nanoparticles to treat human diseases such as breast [4], prostate [5], and lung cancers [6,7]. These treatments employ submicron-sized nanoparticles (3–200 nm) that are synthesized from a variety of materials, including polymers, viral particles, lipids, and even organometallic compounds. This diversity allows for the design and development of nanoparticles that can target, diagnose, and treat disease [6,8,9]. However, most of these nano-therapies produce drug-related side effects or toxicity to non-malignant cells (they are not cell-specific) or are not efficient enough to eradicate the disease. Clearly, there is a need to identify therapeutic approaches that specifically target NSCLC without affecting non-malignant cells. For these reasons, biocompatible nanoparticles are currently being evaluated as a potential alternative for safer and more effective therapeutics. To achieve biocompatibility, the molecule must interact with the cellular environment without inducing high levels of toxicity and immunogenic, thrombogenic, and/or carcinogenic responses [10]. Non-metallic nanoparticles have shown potential as anticancer therapeutics due to their low cytotoxic effects. For example, nanoparticles composed of calcium, such as calcium carbonate, have been shown to reduce the size of solid tumors generated by gastric cancer in a mouse model [11,12]. Other studies have used non-metallic nanoparticles, including sulfur-containing compounds such as NOSH aspirin. These nanoparticles induce apoptosis in several malignant cell lines such as colon cancer cell lines [13], as well as cancer in animal models, by altering cell cycle progression and tumor inhibition [14], without causing significant damage to non-malignant cells [15]. Our previous studies demonstrated the therapeutic potential of CaS nanoclusters based on skin and breast cancer in vitro models [16,17]. Rodriguez-Martinez et al. [16] reported that CaS nanoparticles led to a significant reduction in cancer cell proliferation attributed to dissociation into calcium ions and $H_{2}S$ delivery under acidic environments [16]. However, how CaS nanoclusters enhance their therapeutic effects on cancer cells while minimizing damage to healthy cells remains unknown. This study investigated the role of CaS nanoclusters (1.0 +/− 0.2 nm) in inducing cytotoxicity through DNA damage, oxidative stress, the disruption of the cell cycle, and cellular repair mechanisms, while evaluating their selectivity toward malignant cells over their healthy cell counterparts, lung fibroblasts.

## 2. Results

### 2.1. CaS Nanoclusters Induce Significant DNA Damage in Lung Adenocarcinoma Cells

**Figure 1:**
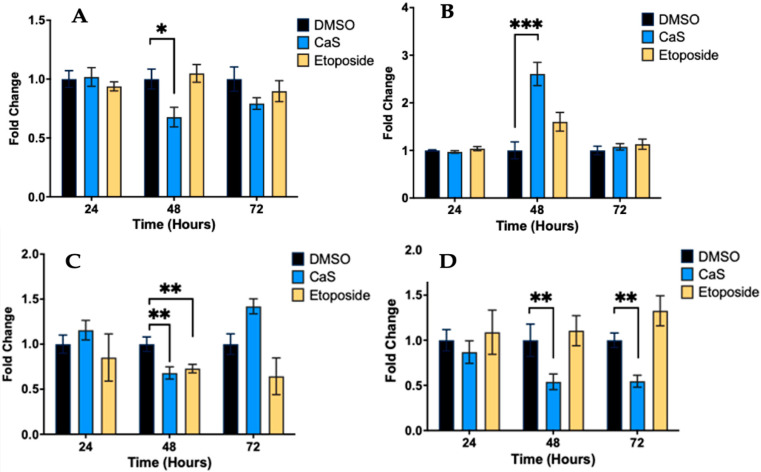
Assessment of DNA damage in malignant lung (HCC827) cells treated with CaS nanostructures. Flow cytometry using directly conjugated antibodies identified cells with no DNA damage (**A**) or double-stranded DNA breaks (**B**); in addition, this assay identified cells with activation of ataxia telangiectasia mutated kinase (ATM) (**C**) and phosphorylated H2a.X (**D**). Data are indicated as mean and standard error of mean (SEM). Statistical significance was set at *p* < 0.05 (***, *p* < 0.002; **, *p* < 0.03; *, *p* < 0.05).

To investigate the effect of CaS nanoclusters on the induction of DNA damage in lung adenocarcinoma, treated cells were analyzed by flow cytometry. Our results showed a significantly lower percentage of cells conserving intact DNA (*p* ≤ 0.05) (Figure 1A) and an increase in the percentage of DNA double-strand breaks (*p* ≤ 0.002) in HCC827 cells treated with CaS at 48 h compared to the vehicle control (Figure 1B). Determined by the dual staining of phosphorylated ATM/H2a.X, this observation is consistent with that for single-positive cells stained for pATM (*p* ≤ 0.03) (Figure 1C) or pH2a.X (*p* ≤ 0.03) (Figure 1D) at the same time point. Notably, we also observed a significant decrease in single-positive pATM cells at 72 h.

### 2.2. CaS Nanoclusters Increase Calcium Concentrations in Lung Adenocarcinoma Cells at Early Time Points, Leading to Cell Death

**Figure 2:**
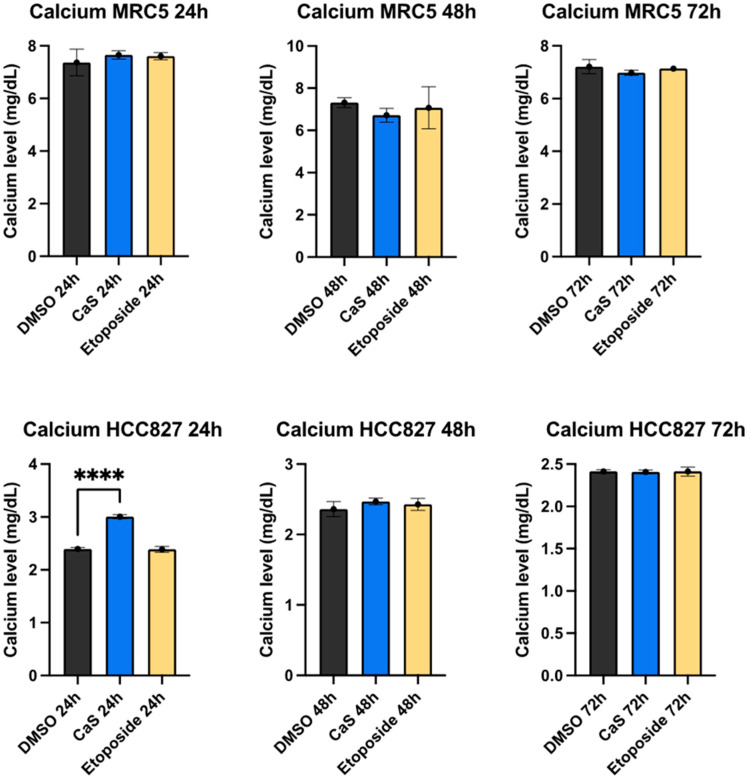
Calcium concentration (md/dL) changes in malignant lung (HCC827) cells treated with CaS nanostructures. Cell supernatants were collected to measure calcium concentration by the o-Cresolphthalein–calcium reaction, which produces a purple complex that absorbs at 595 nm and 405 nm. Data are indicated as mean and SEM. Statistical significance was set at *p* < 0.05 (****, *p* < 0.002).

Increased calcium levels are recognized as a stress response responsible for DNA damage and cellular lytic events. Following our initial observation that CaS nanoclusters induced DNA damage in HCC827 cells, we measured extracellular ${Ca}^{2+}$ concentrations to gain insight into their response to CaS treatments. The baseline calcium level differs between cell lines. Although non-malignant MRC5 presents higher initial physiological calcium levels, these are not affected upon exposure to CaS nanoclusters. In contrast, our results showed that the supernatant of malignant cells treated with CaS nanoclusters has a significantly increased extracellular calcium concentration (*p* < 0.001) after 24 h compared to the DMSO control (Figure 2).

### 2.3. CaS Nanoclusters Trigger Oxidative Stress in Lung Adenocarcinoma Cells

**Figure 3:**
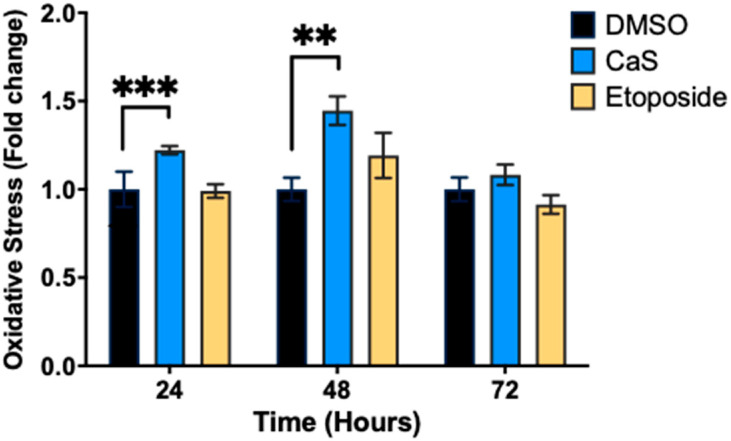
Assessment of oxidative stress in malignant lung (HCC827) cells treated with CaS nanostructures. Flow cytometry was performed to determine the percentage of cells undergoing oxidative stress based on the intracellular detection of superoxide radicals. Data are indicated as mean and SEM. Statistical significance was set at *p* < 0.05 (***, *p* < 0.002; **, *p* < 0.03).

It is well known that oxidative stress and ${Ca}^{2+}$ influx stress responses can lead to DNA damage by generating reactive oxygen species (ROS) [18]. Therefore, our next step was to measure oxidative stress in HCC827 cells after treatment with CaS nanoclusters by flow cytometry. The results show that CaS treatments significantly increased superoxide radicals at 24 and 48 h (*p* < 0.002 and *p* < 0.03, respectively) compared to DMSO controls (Figure 3).

### 2.4. Molecular Mechanisms of CaS Nanoclusters in Lung Cancer Cells Are Less Likely to Be Regulated by Mitochondrial Pathways

**Figure 4:**
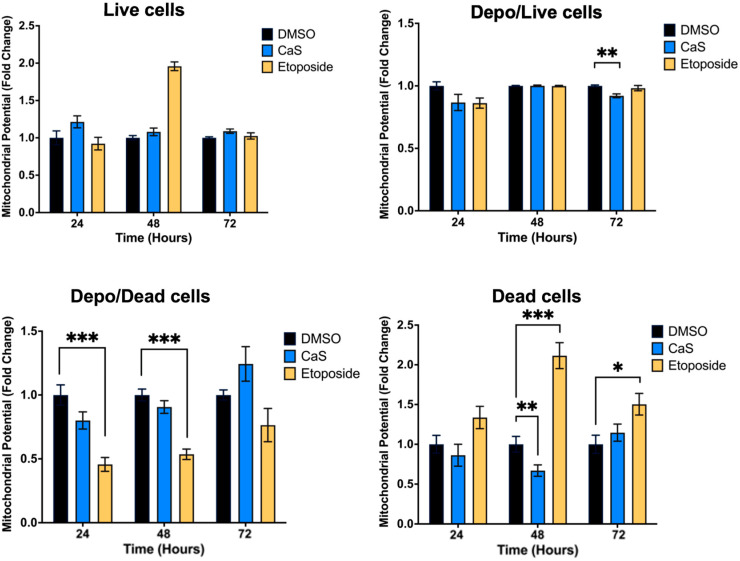
Changes in mitochondrial membrane potential in malignant lung (HCC827) cells treated with CaS clusters. We examined changes in mitochondrial membrane potential as an indicator of mitochondrial dysfunction and cellular health. Cells in depolarized, depolarized/dead, dead, or live states were identified by using a cationic lipophilic dye in combination with dead cell marker 7-AAD in flow cytometry. Data are indicated as mean and SEM. Statistical significance was set at *p* < 0.05 (***, *p* < 0.002; **, *p* < 0.03; *, *p* < 0.05).

**Figure 5:**
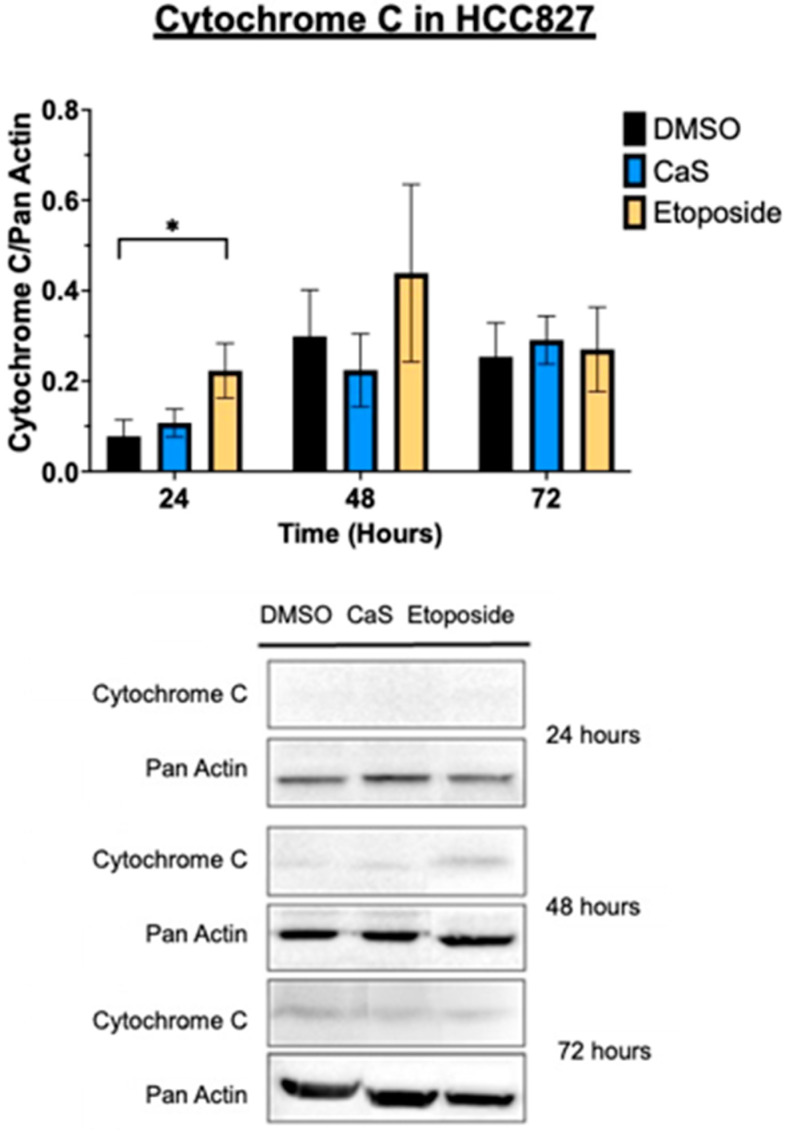
Protein expression analysis of cytochrome C in malignant lung (HCC827) cells treated with CaS clusters. Data are indicated as mean and SEM. Pan Actin was used as a loading control; * *p* < 0.05.

DNA damage can impair mitochondrial function, disrupting the electron transport chain and causing electron leakage, leading to the increased generation of reactive oxygen species. To understand the molecular mechanisms of CaS treatment, we evaluated its effects on the mitochondrial potential of malignant HCC827 cells. Flow cytometry results showed no significant difference in the mitochondrial potential of CaS-treated cells compared to DMSO controls (Figure 4). However, etoposide demonstrated its role via mitochondrial events as described in the literature. Since cytochrome C is released into the cytosol during the initiation of apoptosis via the mitochondrial pathway, we evaluated cytochrome C expression to determine the involvement of CaS in the mitochondrial apoptotic pathway. Cytochrome C was only identified in the HCC827 cell line, although there were no statistically significant changes at any time point between the experimental groups and the control; the etoposide treatment showed the expected increase at all time points (Figure 5).

### 2.5. CaS Nanoclusters Are Less Likely to Affect Intrinsic Apoptotic Pathways

**Figure 6:**
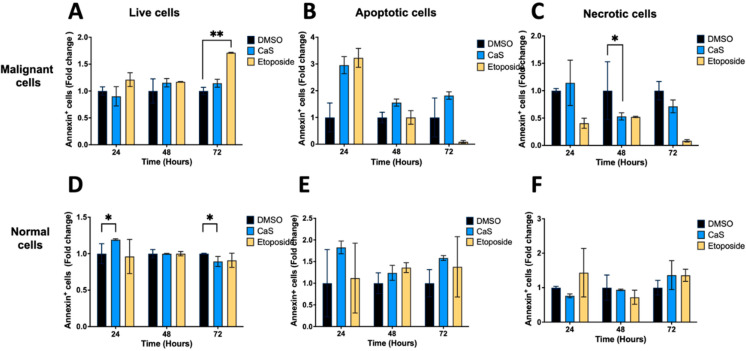
Effects of CaS clusters on Annexin V levels in malignant (HCC827) and normal (MRC5) lung cells. We examined the impact of CaS clusters on human lung cells by measuring Annexin V by fluorescent cell counting at different time points. Live (Panels **A** and **D**), apoptotic (Panels **B** and **E**), and necrotic (Panels **C** and **F**) cells were identified by labeling Annexin V and propidium iodide. Data are indicated as mean and SEM. Statistical significance was set at *p* < 0.05 (**, *p* < 0.03; *, *p* < 0.05).

**Figure 7:**
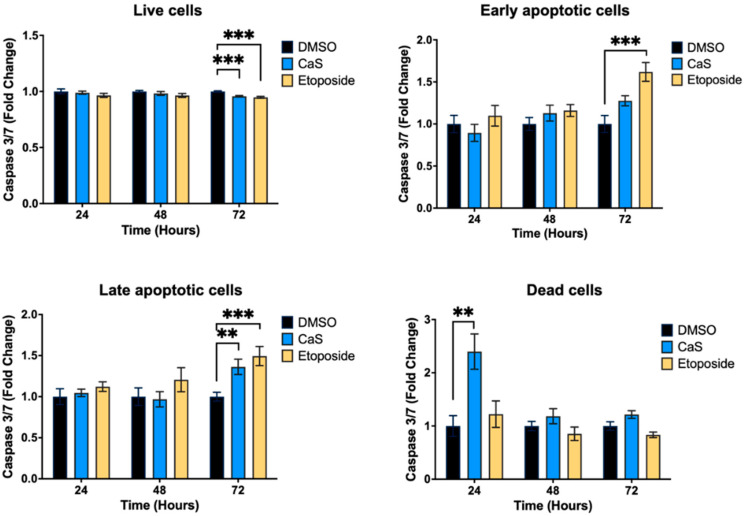
Expression of caspase 3/7 in malignant lung (HCC827) cells treated with CaS clusters. We examined the impact of CaS clusters on the expression of caspase 3/7 by flow cytometry at different time points. Cells in early apoptosis, late apoptosis, live, or dead states were identified by a combination of caspase 3/7 proteolytic activity and dead cell marker 7-AAD. Data are indicated as mean and SEM. Statistical significance was set at *p* < 0.05 (***, *p* < 0.002; **, *p* < 0.03).

Given our preliminary assessment of mitochondrial apoptotic pathways, we evaluated the effects of CaS on alternative apoptotic pathways in malignant lung cells. We evaluated the effects of CaS in the HCC827 and MRC-5 cell lines using Annexin V FITC (Figure 6). We observed no significant difference in the live and apoptotic populations of HCC827 and MRC-5 after treatment compared to DMSO controls. In addition, we evaluated the expression of the apoptotic marker caspase 3/7 in the HCC827 and MRC-5 cell lines at 24, 48, and 72 h after single-dose treatment (Figure 7). When testing for caspase-dependent pathway mediators, we did not observe a significant difference in the expression of caspase 3/7 in the CaS treatment group compared to DMSO, but we did observe the characteristic apoptosis-driven effect of etoposide in malignant cells.

**Figure 8:**
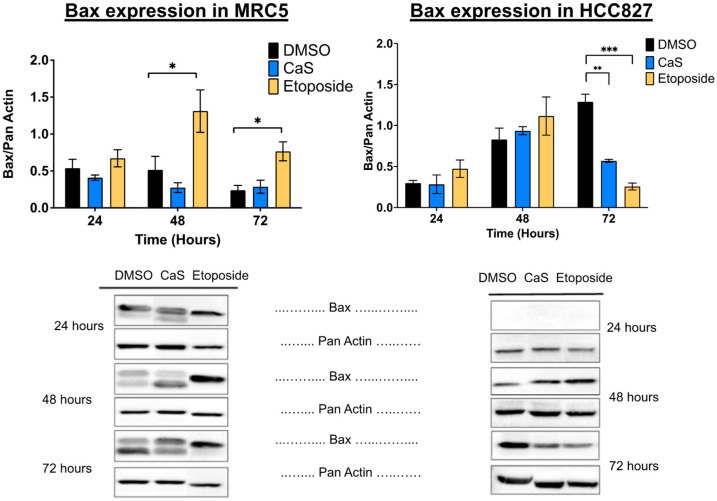
Protein expression analysis of apoptotic marker Bax in normal (MRC5) and malignant lung (HCC827) cells treated with CaS clusters. Data are indicated as mean and SEM. Pan Actin was used as a loading control. Statistical significance was set at *p* < 0.05 (***, *p* < 0.001; **, *p* < 0.03; *, *p* < 0.05).

To examine the protein expression of other apoptotic markers, we also performed immunoblotting on the HCC827 and MRC-5 cell lines at 24, 48, and 72 h after single-dose treatment. The pro-apoptotic protein Bax was significantly decreased in HCC827 treated with CaS at 72 h when compared to DMSO (Figure 8), while in the MRC-5 cell line, when compared to the DMSO control, neither caspase 3 nor Bax showed significant changes in protein expression.

**Figure 9:**
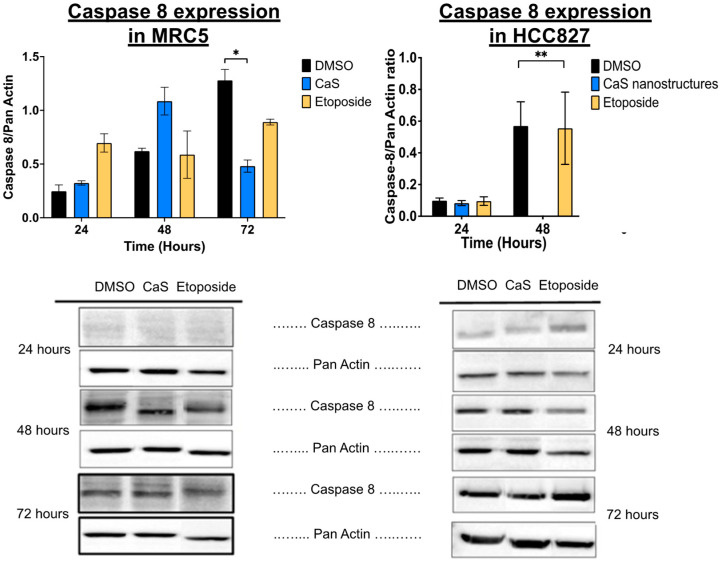
Protein expression analysis of caspase 8 in normal (MRC5) and malignant lung (HCC827) cells treated with CaS clusters. Data are indicated as mean and SEM. Pan Actin was used as a loading control. Statistical significance was set at *p* < 0.05 (**, *p* < 0.03; *, *p* < 0.05).

**Figure 10:**
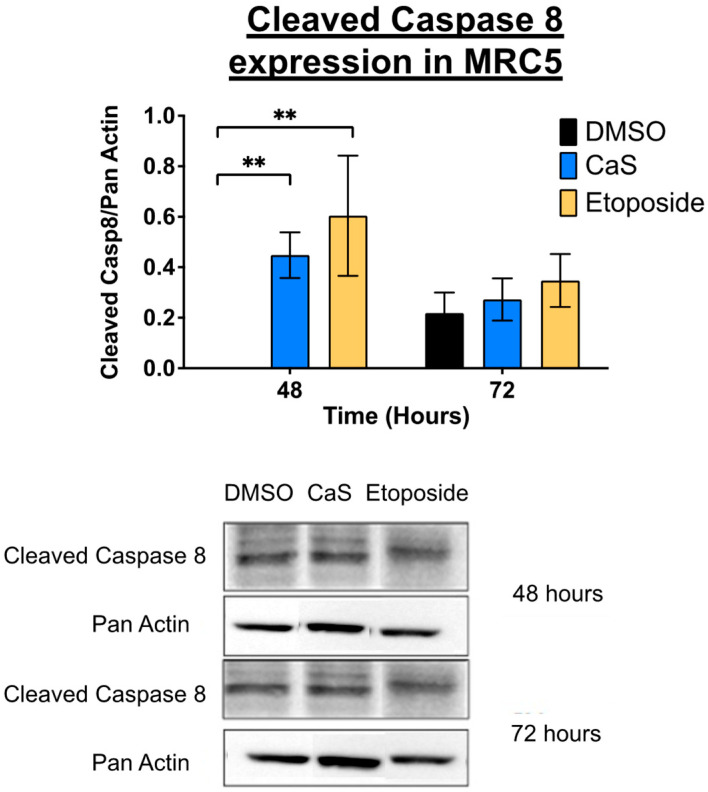
Protein expression analysis of cleaved caspase 8 in normal (MRC5) lung cells treated with CaS clusters. Data are indicated as mean and SEM. Pan Actin was used as a loading control. Statistical significance was set at *p* < 0.05 (**, *p* < 0.03).

Separate studies showed that the apoptosis initiator caspase 8 decreased significantly at 72 h in CaS-treated malignant HCC827 compared to MRC-5 cells (Figure 9), while the cleaved form of the enzyme was detected and increased at 48 h, although this was not significant (Figure 10).

### 2.6. CaS Nanoclusters Selectively Arrest Lung Adenocarcinoma Cells but Not Healthy Cells

**Figure 11:**
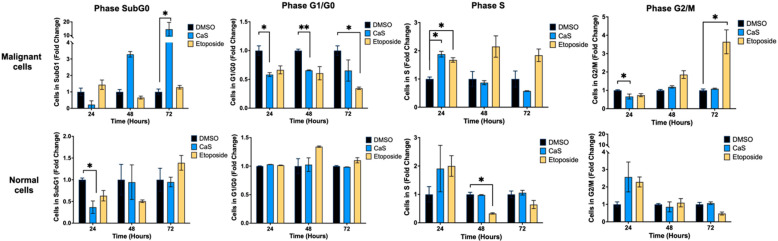
Changes in cell cycle phases in lung cells treated with CaS clusters. We examined the impact of CaS clusters on the cell cycle phases of human lung cells by measuring DNA content at different time points. Cells in resting (SubG0), normal growth (G1/G0), DNA synthesis (S), and growth/mitosis (G2/M) phases were identified after determining the proportion of cells in each stage of the cell cycle based on variations in DNA content. Data are indicated as mean and SEM. Statistical significance was set at *p* < 0.05 (**, *p* < 0.03; *, *p* < 0.05).

DNA damage and oxidative stress are known to affect the cell cycle through the activation of various signaling pathways and checkpoints. Therefore, we investigated the effect of CaS on the cell cycle to elucidate the molecular mechanisms of its anticancer therapeutic effects. Flow cytometry results (Figure 11) showed that CaS nanoclusters at 24 and 48 h significantly (*p* < 0.05) reduced the population of malignant (HCC827) cells in the G0/G1 phase compared to DMSO. The G0/G1 phase is characterized by cell growth and preparation for DNA replication, suggesting that CaS disrupts DNA replication in malignant cells. The population of malignant cells in the S phase increased significantly (*p* < 0.05) only at 24 h after CaS treatment. The S phase is critical for DNA replication, and flow cytometry results suggest that CaS treatment induces replication stress, causing cells to accumulate in the S phase at 24 h due to checkpoints that inhibit progression to the G2/M phase. Over time, this could result in replication fork collapse, leading to double-strand DNA breakage which results in cell death [19]. The cells treated with etoposide showed an increase in cells in the S and G2/M phases, as expected. This observation correlates with a significant decrease (*p* < 0.05) in the malignant cell population in the G2/M phase at 24 h. Normal lung cells did not show any significant changes in cell cycle after CaS treatment other than a transient decrease at 24 h at the sub-G0 phase.

**Figure 12:**
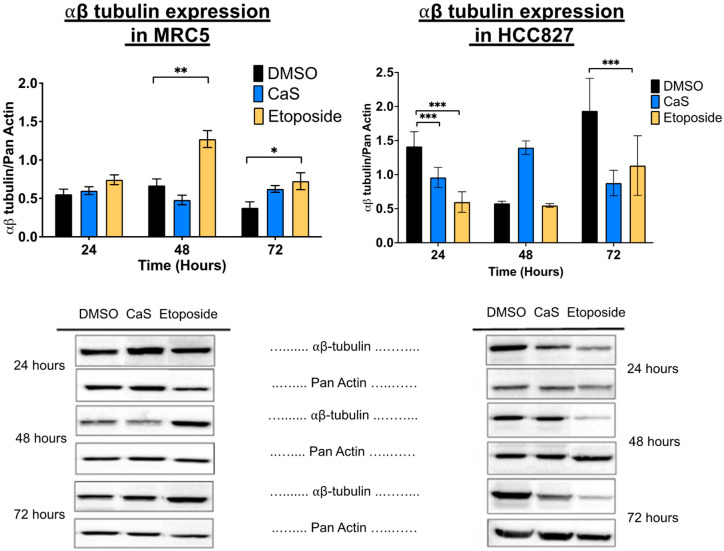
Changes in expression of αβ-tubulin in lung cells treated with CaS clusters. We examined the expression of αβ-tubulin by Western blot in normal (MRC5) and malignant lung (HCC827) cells. Data are indicated as mean and SEM. Statistical significance was set at *p* < 0.05 (***, *p* < 0.002; **, *p* < 0.03; *, *p* < 0.05).

Following protein analysis, we observed that in the HCC827 cell line, αβ-tubulin expression was significantly decreased in the presence of CaS at 24 h compared to DMSO (Figure 12), and the MRC-5 cells showed a significant increase in this protein’s expression at 72 h in the CaS-nanostructure-treated cells when compared to DMSO. This outcome suggests that at these specific time points, CaS nanostructures act as a microtubule inhibitor in malignant cells. Finally, CaS significantly (*p* < 0.05) increased the population of malignant (HCC827) cells in the sub-G0 phase at 72 h compared to DMSO. During the sub-G0 phase, cells undergo DNA fragmentation and eventually die. At the same time, CaS significantly decreased the normal (MRC5) lung cell population after 24 h of treatment, suggesting that CaS favors normal cell replication or that the effect is transient.

## 3. Discussion

The effects of calcium and sulfur as a treatment for several cancers have been studied previously [12,15,20,21], including CaS nanostructures [16,17]. Previous works focused on the effects of CaS nanostructures on the viability and cell cycle of breast cancer cells and cultures of predominantly mesenchymal melanoma cells. In the latter, it was found that the clusters facilitated vinculin delocalization in the intracellular matrix, a result associated with the regulation of focal adhesion kinase (FAK). The regulation of FAK was proposed to activate classical apoptotic death pathways that result in malignant cell death. However, none of these studies addressed the effects of NSLCC or the biological mechanisms associated with their respective observations.

Rivera [22] and Rodriguez [16] have also pointed out the importance of pH in the chemical processes that are intrinsically related to CaS chemistry. CaS readily dissociates in acidic media to form gaseous $H_{2}S$ as well as $H_{2}S$- HS-, and $S_{2}$ species in the solution. An extracellular pH is a landmark of the most malignant tumors: this results from the high metabolic rate of proton production and export from the intracellular fluid to the extracellular matrix [16]. The species in a solution are believed to actively participate in chemical processes that result in the activation of malignant cells. Rodriguez and coauthors [16] have pointed out the dephosphorylation of mutated FAK in tyrosine 397. This follows from a comparison of classical dephosphorylation mechanisms mediated by cysteine residues in benign cells.

This report is the first to focus on the biological mechanisms associated with the effect of CaS nanoparticles in NSCLC, demonstrating that CaS nanoclusters exert a multifaceted negative impact on NSCLC proliferation and progression, making them ideal for consideration as a potential treatment. We systematically explored biologically viable mechanisms of action to support previous observations, specifically of CaS nanostructures and cancer, published by our group [16,17].

We first examined DNA damage in NSCLC cells after a single dose of CaS nanoclusters, and there was a significant increase in DNA double-strand breaks combined with a significant decrease in intact DNA and single-positive pATM and pH2a.X cells, suggesting that CaS nanoclusters induce DNA damage, which can lead to cell death (Figure 1). In addition, there was a significant reduction in pATM at 72 h, which correlates with the observed increase in sub-G0 cells and late apoptotic cells, which do not require the activation of the DNA repair machinery [23].

The ability of CaS nanoparticles to dissociate into calcium ions (${Ca}^{2+}$) and sulfides under acidic conditions, such as the tumor microenvironment discussed in the literature [16,17,20], is important. We analyzed the extracellular calcium levels in a supernatant of malignant and non-malignant cells treated with CaS nanoclusters. The malignant HCC827 cells showed a significant increase in extracellular calcium concentration. We proposed that CaS dissociation increases extracellular ${Ca}^{2+}$, leading to intracellular dysregulation and cell necrosis. This releases intracellular calcium into the microenvironment, further increasing extracellular concentrations (Figure 2). Another possible mechanism associated with the anticancer effect of sulfur-based nanoparticles (SBNPs) is that nanoparticle accumulation increases the permeability of cancer cells and, in this case, leads to ${Ca}^{2+}$ leakage into the cell from CaS dissociation, resulting in cancer cell death [22]. In addition, ${Ca}^{2+}$ dissociated from the CaS nanoclusters can also react with proteins and cause thermodynamic changes that may lead to cell death [24,25]. We ruled out the spontaneous dissociation of CaS nanoclusters in the media as the source of calcium by measuring basal ${Ca}^{2+}$ levels in the corresponding media and control wells without cells. Using these controls, we attributed CaS dissociation to the acidic conditions of the microenvironment, as described by Rodriguez et al. [16]. In another study, Cárdenas et al. [26] reported that other cancer cell lines such as breast (MCF-7) and prostate cancer-derived (PC-3) tumorigenic cell lines showed that, when exposed to a high concentration of ${Ca}^{2+}$, a deficient transfer between the ER and mitochondria could promote a bioenergetic crisis. This event can lead to necrotic death by mitotic catastrophe as cells proceed through mitosis, as shown in our results.

Furthermore, we found that CaS nanoclusters induced oxidative stress in lung adenocarcinoma cells, as evidenced by the increased levels of reactive oxygen species (Figure 3). Together, these events may contribute to the accumulation of genetic mutations and promote selective cancer cell death. The results were validated also by the absence of significant oxidative stress in the etoposide control, since this is not its mechanism of action.

Interestingly, our data demonstrate that the molecular mechanisms of CaS nanoclusters are less likely to involve mitochondrial pathways and that they trigger alternate mechanisms that induce alterations in cell cycle checkpoints. We found no significant changes in mitochondrial membrane potential in live cells (Figure 4) or in cytochrome C release (Figure 5), suggesting that the observed effects are independent of mitochondrial electron transport chain events. Moreover, the apoptotic pathways appear to be minimally affected by CaS treatments, as these treatments did not alter key apoptotic biomarkers such as Annexin V (Figure 6), caspase 3/7 activity (Figure 7), or Bax (Figure 8). Changes in other molecules, like caspase 8 inhibition, may be involved in the stability of tubulin during alternate cell death pathways, which include necroptosis and mitotic catastrophe [27]. In this study, we showed that the studied caspases are not activated when exposed to CaS at any time point, and their cleaved or activated versions are not observed (Figure 9 and Figure 10). CaS affects the HCC827 cell cycle by stagnating these cells in the G1/G0 phase and preventing their entry to the S phase (Figure 11). Our results point to classic mitotic catastrophe markers in the HCC827 cell line due to the cellular stress induced by CaS. This pathway is characterized by the apparent lack of activation of key caspases, such as caspase 3 and caspase 7, during cell death, eventually affecting the cell cycle by interrupting the synthesis of microtubules (Figure 12) in the G1/S phase.

We showed that CaS nanoclusters selectively arrest lung adenocarcinoma cells at specific points in the cell cycle without affecting the proliferation of normal lung fibroblasts. These data are consistent with our previous studies [17] in that CaS nanoclusters affect the cell cycle specifically at G0 and G0/G, but this time, we also observed arrest at the S phase. These results suggest that CaS may act as a microtubule inhibitor due to cell arrest in the S phase. For the MRC-5 cell line, cell population at the sub-G1 stage decreased significantly at 24 h when compared to the control, and an increase in cell population at the G2/M phase was exhibited. This finding correlates with previous studies reporting that etoposide is more effective in arresting cells at the S and G2 phases [28,29]. The use of etoposide as a positive control validated the obtained results for CaS nanoclusters in malignant cells arrested at the S phase.

## 4. Materials and Methods

### 4.1. Cell Culture and Positive and Negative Controls

We cultured the human epithelial non-small-cell lung adenocarcinoma (NSCLC) HCC827 (ATTC) and normal lung fibroblast MRC-5 (ATCC) cell lines under standard conditions at 37 °C, 5% CO_2_, and 98% relative humidity. The HCC827 cells were maintained in an RPMI-1640 medium (ATCC). The MRC-5 cells were cultured in the Eagle’s Minimum Essential Medium. Both media were supplemented with 10% fetal bovine serum (FBS) and 1% penicillin–streptomycin and were replaced every 48 h. Etoposide, a Topoisomerase II inhibitor used as a common cancer treatment, was used as a positive control and DMSO as a control vehicle in all experiments.

### 4.2. Synthesis of Calcium Sulfide (CaS) Nanoclusters and Cell Treatments

Nanostructures were synthesized as described in patent #US 8,945,494 B1 [30]. We extended a method employed earlier by our group to prepare cadmium sulfide (CdS) nanostructures to the preparation of calcium sulfide (CaS) nanoclusters. In this study, the flasks containing the cell cultures were randomly assigned to one of three treatment groups: dimethyl sulfoxide (DMSO) vehicle (2%), CaS nanoclusters (3.8 µM of the synthesized particles based on 100% calcium conversion during synthesis), or etoposide (10 µM). The DMSO and etoposide concentrations were selected based on the literature [31]. Samples were collected at 24, 48, and 72 h after single-dose exposure to the corresponding treatments.

### 4.3. Annexin V FITC and Cell Cycle Assays

We assessed apoptosis using 1 × 10^5^ of each cell type to run Annexin V FITC/PI apoptosis assays (Nexcelom Bioscience, Lawrence, MA, USA). We used 1 × 10^6^ of each cell type to study the cell cycle using the PI Cell Cycle kit (Nexcelom Bioscience). Cell suspensions were analyzed using a Cellometer K2 cytometer and De Novo FCS Express 5.0 software.

### 4.4. Whole-Cell Protein Extractions and Immunoblotting

Following trypsinization, cells were lysed in the presence of protease inhibitor cocktails (Sigma-Aldrich, St.Louis, MO, USA) for 30 min. Protein concentrations were determined using BCA Protein Assay kits (Pierce Biotechnologies, Rockford, IL, USA). For protein expression analyses, all reagents were bought from Bio-Rad (Hercules, CA, USA) unless otherwise specified. We used Mini-PROTEAN TGX pre-casted 4–20% acrylamide gels. The proteins were transferred to polyvinylidene fluoride (PVDF) membranes. All antibodies were obtained from Cell Signaling Technologies (Danvers, MA, USA) unless otherwise specified. Primary antibodies were diluted in wash buffer containing 5% BSA, and secondary antibodies were diluted in a solution containing 5% non-fat milk, a 1:1000 anti-biotin HRP-linked antibody to detect the biotinylated protein ladder, and goat anti-rabbit IgG-peroxidase antibodies (Sigma-Aldrich). Antibodies were used with T-PBS washing buffer as a diluent: caspase 8 (1:500, Cell Signaling technologies, Cat. #9746), cytochrome C (Cyto-C) (1:1000, Cell Signaling Technologies, Cat. #11940), alpha-beta (αβ)-tubulin (1:2000, Cell Signaling Technologies, Cat. # 2148), the Bax antibody (1:500, Cell Signaling Technologies, Cat. # 5023), and the Pan Actin antibody (1:2000). Targeted proteins were analyzed using the Azure c600 imaging system by exposure to chemiluminescent substrate Radiance Plus (Azure Biosystems, Dublin, CA, USA). Densitometry analyses were performed using Quantity One 1-D analysis software (Bio-Rad). Membranes were exposed to Restore Western Blot Stripping Buffer (Thermo Scientific, Waltham, MA, USA) for one hour at 50 °C followed by a 2 h blocking step with 5% non-fat milk for re-probing as needed.

### 4.5. Extracellular Calcium Free Ion Assay

Cell supernatants were collected at each time point to measure extracellular calcium ions using the Calcium Assay Kit according to the manufacturer’s protocol, using the optical density at 595 nm in a MultiSkan FC microplate spectrophotometer (Thermo Scientific). Samples were normalized to supplement growth media.

### 4.6. Assays Using Flow Cytometry

Flow cytometry assays were conducted using the Muse Cell Analyzer (EMD Millipore) and the Muse Oxidative Stress, Caspase 3/7 Assay, MitoPotential, and Multi-Color DNA Damage kits. Each sample was prepared according to manufacturer’s protocol in triplicates and measured at 24, 48, and 72 h after the addition of DMSO, CaS nanoclusters, and etoposide.

### 4.7. Statistical Analysis

Using IBM SPSS software Version 29, normality tests, namely, the Shapiro–Wilk and Kolmogorov–Smirnov tests, were performed to determine the distribution of the data. ANOVA was used to determine data variance, and post hoc analysis was used to determine differences between groups (control vs. experimental). The Kruskal–Wallis was also used to determine distribution, and the Mann–Whitney U test was used to determine differences between groups. The significance value considered was *p* ≤ 0.05. The data were represented as means ± SEMs (standard error of means) or the fold change, as applicable. Analyses were performed using SPSS v 23.0 (IBM Corporation). Graphical representations were constructed using GraphPad Prism 10.2.3 software.

## 5. Conclusions

**Figure 13:**
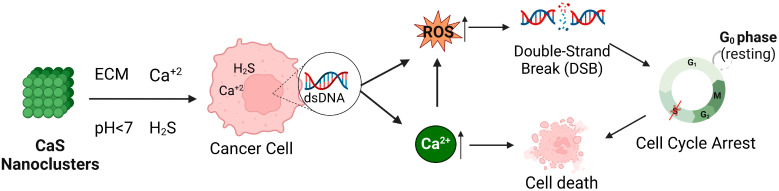
Hypothetical model of the effects of CaS nanostructures on cellular pathways. This schematic illustration demonstrates how selective interactions between CaS nanostructures and the acidic extracellular microenvironment (ECM) of cancer cells trigger the release of hydrogen sulfide (H_2_S) and calcium ions (Ca^2+^). The influx of calcium ions into the cell increases intracellular Ca^2+^ levels and, in addition, generates reactive oxygen species (ROS), which can lead to DNA damage through double-strand breaks (DSBs). Simultaneously, this increase in intracellular Ca^2+^ contributes to cellular stress and potential damage mechanisms, which lead to a decrease in the G1/G0 phase, preventing the cells from completing the S phase after 24 h and progressing to G2/M.

This is the first study that addresses the biological mechanisms of the selective actions of CaS nanoclusters in cancer cells. Selective induction by the acidic malignant cells’ microenvironment promotes CaS dissociation and cell death, including mitotic catastrophe and necroptosis simultaneous events. Based on our observations, we propose a hypothetical model (Figure 13) in which CaS induces cell arrest in the G0, G0/G, and S phases in HCC827 cells, which is related to DNA-damage-induced cell death, while MRC-5 cells remain unaffected. However, in this study, we observed that HCC827 cells exhibited an increase in superoxide radicals and in DNA double-strand breaks at 48 h after the addition of CaS nanoclusters. These findings suggest that CaS nanoclusters may offer a novel and selective approach to the treatment of NSCLC by taking advantage of the inherent vulnerability of cancer cells while sparing non-malignant cells. Future studies are warranted to determine whether the effects of CaS nanoclusters are reproducible in other cancer types through similar mechanisms.

## Data Availability

The data generated in this study are available within the article.
